# Mechanism of piperine in affecting apoptosis and proliferation of gastric cancer cells via ROS‐mitochondria‐associated signalling pathway

**DOI:** 10.1111/jcmm.16891

**Published:** 2021-08-31

**Authors:** Li Guo, Yi Yang, YongJia Sheng, Jin Wang, Shuiliang Ruan, Chenyang Han

**Affiliations:** ^1^ Department of Center Laboratory The Second Affiliated Hospital of Jiaxing University Jiaxing China; ^2^ Department of Pharmacy The Second Affiliated Hospital of Jiaxing University Jiaxing China; ^3^ Department of Gastroenterology The Second Affiliated Hospital of Jiaxing University Jiaxing China

**Keywords:** apoptosis, gastric cancer, mitochondria, piperine, ROS

## Abstract

Piperine (PIP), the main active ingredient in pepper, belongs to the cinnamamide alkaloid. PIP has been found to have functions, including anti‐oxidation, immune regulation, anti‐tumour and promotion of drug metabolism. The present study was mainly designed to reveal the anti‐tumour effect of PIP against gastric cancer and the relevant mechanism. In brief, the undifferentiated human gastric cancer cell HGC‐27 was used, which was treated with different concentrations of PIP. As a result, PIP could inhibit proliferation and induce apoptosis of HGC‐27 cells in a dose‐dependent manner. The mechanism of PIP was associated with ROS increase and mitochondrial damage, simultaneously, the expression of key proteins of apoptosis was affected, including Bcl‐2, Bax, Cyt‐c, Caspase‐9 and Caspase‐3. Pre‐treatment of ROS scavenger NAC HGC‐27 cells could significantly reduce PIP‐induced apoptosis and inhibit the activation of apoptotic signals. Consistently, PIP could induce ROS to increase and activate apoptotic signals in the animal model. Therefore, the present study showed that PIP can induce the generation of ROS, thereby promoting the activation of mitochondrial apoptotic pathway and exerting anti‐tumour effects.

## 1 INTRODUCTION

Piperine (PIP) is an active alkaloid mainly extracted from pepper, with a variety of physiological and pharmacological activities.^1^ Moreover, PIP has a broad market and considerable economic benefits in China. Extraction of pepper is the main way to obtain PIP. There are three main types of extraction methods, including acid extraction method (PIP is a basic substance^2^, ^3^). Pharmacological studies have confirmed that PIP has a series of functions, including analgesia, antipyresis, anti‐oxidation, anti‐tumour, immune regulation, etc.^4^, ^5^ In the study of the anti‐tumour activity of piperine, several cancer models showed cytotoxic and inhibitory effects on a variety of cancer cell lines, including colon cancer, lung cancer, breast cancer, pancreatic cancer, kidney cancer and prostate cancer.^6^ In addition, anti‐tumour activity has also been found in various animal models. Piperine triggers cell death by causing cell apoptosis and necrosis. The molecular apoptotic effects of piperine activity include reduction of cell lymphoma, caspase‐3, poly(ADP‐ribose) polymerase(PARP) activation, oncogene and NH2‐terminal kinase activation are involved. In addition, piperoning was found to restore the normal function of the mutated p53 protein in human colon cancer cells, and piperoning can also induce cell death through autophagy.^7^ Although many studies have proved that PIP has an anti‐tumour effect and can exert its effect through a variety of mechanisms. However, there are few studies on PIP in anti‐gastric cancer, and its exact mechanism still needs to be further studied.

Gastric cancer is one of the most common gastrointestinal malignancies globally. Epidemiological statistics show that the incidence of gastric cancer increases with ageing, reaching a peak at 50–70 years old, and the incidence in female is higher than that in male.^8^ The pathogenesis of gastric cancer is closely associated with Helicobacter pylori infection and gastric ulcer. Meanwhile, the contamination and contact of nitrogen nitroso compounds, polycyclic aromatic hydrocarbons and heterocyclic amine compounds can also cause gastric cancer. Surgical resection and chemotherapy have become the main therapeutic approaches for gastric cancer.^8^ However, there are still certain gaps in the long‐term intervention of prevention and prognosis of gastric cancer. Pepper, as the main seasoner for daily intake, plays a role in a variety of diets. Therefore, based on the previous findings, it is speculated that PIP may have a certain anti‐tumour effect on gastric cancer. Therefore, we further investigated the anti‐tumour effect of PIP on gastric cancer and the relevant mechanism.

## 2 MATERIALS AND METHODS

### 2.1 Cell culture and grouping

Undifferentiated human gastric cancer cell line HGC‐27 (cryopreservation in the laboratory) was cultured at 37°C in an incubator containing 5% CO_2_. When cells reached 80% confluency, cells were digested with 0.25% trypsin, adjusted to 5 × 10^5^/ml with 1640 medium containing 20% foetal bovine serum (FBS), and then inoculated into the culture plate. Cell growth was observed under an inverted microscope. When cells grew into the logarithmic phase, cells were harvested into prepare cell suspension. After adjusting the cell concentration, cells were divided into control group (Con group) and PIP group. Cells in Con group were conventionally cultured, According to the pre‐experiment of drug concentration, we set low, medium and high concentrations for the study. PIP groups were treated with PIP at final concentration of 10, 20 and 40 mg/L, respectively.

### 2.2 Cell viability using CCK‐8 assay

Cells in logarithmic phase were harvested adjusted to cell density of 2–5 × l0^5^/ml. Then, cell suspensions of each group were inoculated into 96‐well plates at 100 μl per well at 37°C in an incubator containing 5% of CO_2_. Three parallel wells were set in each group and blank control wells were also set. After replacing 100 μl of fresh medium, PIP was added for incubation for 6, 12, 18, 24, 48 and 72 h. After incubation for indicated time point, 10 μl of CCK‐8 solution was added to each well, followed by additional incubation for 1–4 h. Finally, the absorbance was measured using a microplate reader at 450 nm (A value). Inhibition rate of cell proliferation = (A value of Con group − A value of PIP group)/A value of Control group × 100%.

### 2.3 Intracellular reactive oxygen species (ROS) levels detected by DCFH‐DA Reactive Oxygen Species Assay Kit

HGC‐27 cells in logarithmic phase were digested and adjusted to cell density of 2 × 10^5^/mL and seeded into 6‐well plates. After the incubation for 2–4 h to allow cell adherence, culture medium was changed into serum‐free medium and cultured for 24 h. Afterwards, cells were treated with PIP in 1640 medium containing 20% FBS for 24 h. DCFH‐DA (Abcam) was used to detect intercellular ROS levels. In brief, after the completion of cell culture, cells were replaced with colourless 1640 medium, followed by staining with DCFH‐DA (50 μM) for 30 min. Inverted fluorescence microscope was used for observation and photographing. ROS was stained as green fluorescence. The number of positive staining cells was counted, and the absorbance was measured by a fluorescence spectrophotometer.

### 2.4 Mitochondrial staining in viable cells using Mito‐Tracker green fluorescent mitochondrial probe

HGC‐27 cells in logarithmic phase were harvested adjusted to cell density of 2 × 10^5^/mL, and seeded into 6‐well plates. After incubation for 2–4 h to allow cell adherence, culture medium was changed into serum‐free medium and cultured for 24 h. Mito‐Tracker Green probe was used to detect mitochondrial staining. Cells were first changed to colourless 1640 medium, and probes were diluted at a ratio of 1:500. After staining for 30 min, cells were observed and photographed under an inverted fluorescence microscope. Mitochondria of viable cells were stained as green fluorescence, and the number of positive staining cells was counted.

### 2.5 Detection of mitochondrial membrane potential

Rhodamine mitochondrial membrane potential detection kit (Bestbio, Shanghai, China) was used in this assay. HGC‐27 cells in logarithmic phase were digested, adjusted to cell density at 2 × 10^5^/mL and seeded into 6‐well plates. After incubation for 2–4 h to allow cell adherence, culture medium was changed into serum‐free medium and cultured for 24 h. Afterwards, cells were treated with PIP in 1640 medium containing 20% FBS for 24 h. After the completion of the culture, 10 μM of Rhodamine 123 was added for incubation for 30 min after changing culture medium. Cells were washed twice with PBS, followed by measurement of absorbance using a fluorescent microplate reader. The Con group was used as control and results were shown in percentage.

### 2.6 Detection of apoptosis

Annexin‐FITC and Propidium Iodide double staining kit (BD, USA) was used. Briefly, HGC‐27 cells in logarithmic phase were adherently cultured and treated with PIP in 1640 medium containing 20% FBS for 24 h. Afterwards, cells were collected (including suspended cells), washed three times with PBS, centrifuged at 1500 rpm. After removing the supernatant, cells were resuspended with 200 μl of binding buffer in a flow tube, followed by the addition of 10 μl of Annexin‐FITC was added and 5 μl of Propidium Iodide. Cells were incubated in the dark for 15 min, and flow cytometry was used for detection at the wavelength of Ex = 488 nm and the emission wavelength Em = 530 nm.

### 2.7 Detection of cell cycle

Propidium Iodide staining kit (BD, USA) was purchased. Cell culture method and treatment approach were consistent with the above apoptotic detection. Cells were resuspended in 200 μl binding buffer and then stained with 5 μl of Propidium Iodide. After incubation in the dark for 15 min, cell cycle was determined by flow cytometry.

### 2.8 The expression level of mitochondrial apoptosis‐associated protein using Western‐Blot assay

HGC‐27 cells in logarithmic phase were adherently cultured, treated with PIP in 1640 medium containing 20% FBS for 24 h. Cells were collected, including suspended cells, washed twice with PBS, added with 1.0 ml of RIPA lysate (Beyotime Biotechnology Co., Ltd., Shanghai, China). Cells were lysed on ice for 30 min, centrifuged at 10,000 ***g*** for 15 min, and the supernatant was collected for detection of protein concentration using BCA kit (Beyotime Biotechnology Co., Ltd.). According to the molecular weight, 8–12% SDS‐PAGE gel should not be separately configured. According to the molecular weight, 8–12% SDS‐PAGE gel was separately collocated. The 5x loading buffer was used to supplement the protein sample to 20 μl. Protein samples were boiled for 8min, underwent electrophoresis at 80 V, which subsequently converted into 120V, transferred to PVDF membrane under 300 mA constant for 0.5–2 h. The PVDF membrane was blocked in 5% non‐fatty milk powder for two hours. TBST was used to dilute primary antibodies, including Bax, Bcl‐2, Cyt‐c, Caspase‐3 and Caspase‐9 into 1:800, 1:600, 1:400, 1:500, 1:500 (Abcam), respectively. After incubation with primary antibody, the PVDF membrane was washed twice with TBST, followed by reaction with horseradish peroxidase‐labelled goat anti‐rabbit secondary antibody (Abcam) (dilution 1:20,000). After the incubation, the chemiluminescence method was used to detect the optical density by Image Pro‐Plus 6.0 software. GAPDH was used as an internal control, and the results were shown as a comparison of the optical density values between the target protein and the internal control.

### 2.9 Construction of tumour‐bearing mouse model of gastric cancer

Cell suspension was prepared by collecting HGC‐27 cells in logarithmic phase. 0.3 ml of 0.9% saline solution was added to make the number of viable cells in 10^6^–10^7^ in the centrifuge tube. BALB/c nude mice, male, 6–8 weeks old, weighed 20–23 g were used. Mice were housed in aseptic environment. In the ultra‐clean platform, nude mice were partially sterilized with iodine and alcohol, and cells from the EP tube were extracted with a sterile syringe, then subcutaneously inserted into mice. After the injection was completed, the injection position was gently pressed to prevent the liquid from seeping out. The mice were divided into four groups (10 mice in each group), namely the Con and PIP groups. Tumour‐bearing mouse models in the four groups were constructed using the above method. Mice in the PIP groups were Oral gavaged with PIP at doses of 10, 20, 40 mg/kg, respectively, while mice in the Con group were administered with normal saline once daily for one week.

Animal experiments have been reviewed and approved by the Jiaxing University ethics committee. The entire animal experiment conforms to the relevant regulations of animal ethics and welfare, and the whole process conforms to ethical norms.

### 2.10 Detection of the level of ROS in tumour tissues by viable tissue ROS primary fluorescence assay kit

Viable tissue ROS primary fluorescence assay kit was purchased (Shanghai Dailao Biotechnology Co., Ltd.). Briefly, 200 mg of tumour tissue from mice was isolated, cleaned by reagent A. Afterwards, tissue was aseptically cut, added into the dilution C for shaking, followed by addition into the pre‐cooled DOUNCE homogenizer. The homogeneous tissue was transferred to the conical tube. 50 μl of the homogenate was taken, and 50 μl of the dilution C was added. After adding 20 μl of the staining solution, subsequent mixture and incubation for 20 min, fluorescent microplate reader was used for detection with an excitation wavelength of 490 nm and an emission wavelength of 520 nm.

### 2.11 Detection of the expression of key protein of apoptosis caspase‐3 and Caspase‐9 using immunohistochemical (IHC) staining

The paraffin‐embedded tumour tissues were sliced into 4 μm‐thick sections, baked at 60°C for 2 h, dewaxed in xylene for three times (5 min each), soaked in absolute ethanol for 5 min, then soaked in 95% ethanol twice with (2 min each), soaked in 85% ethanol once for 2 min, rinsed with tap water for 5 min and finally rinsed with distilled water for 3 min. The sections were then placed in 0.01 mol/L citrate buffer (pH = 6.0) and retrieved in the microwave at 98°C for 20 min. It was cooled at room temperature for 30 min and then rinsed with distilled water. Sections were incubated with 3% hydrogen peroxide at room temperature for 10 min to eliminate endogenous peroxidase. Each section was blocked with 2% bovine serum albumin (BSA) at 37°C for 30 min, to prevent non‐specific binding of antigen to the antibody. After discarding BSA, primary antibody was added dropwise for incubation at 37°C for 2 h. The primary antibodies included Caspase‐3 and Caspase‐9 (ABR, USA) (dilution 1:500). Sections were washed with TBS three times for 5 min each, followed by incubation with appropriate secondary antibody at 37°C for 15 min, and subsequent incubation with peroxidase‐labelled streptomycin (Maixin Biotechnology Development Co., Ltd., Fuzhou, China) for 15 min. Then, the sections were rinsed with PBS 3 times for 5 min each. Each slice was added with freshly prepared DAB solution for visualization (DAKO) under microscope for judging the termination of reaction, when the staining was moderate. The sections were rinsed thoroughly with tap water, counterstained with haematoxylin, and sealed. TBS was used as negative control for primary antibody incubation. All sections were photographed and analysed under Olympus‐BX51 upright microscope with Olympus‐DP72 image acquisition system and CRi Nauance multispectral imaging system (Cambridge Research & Instrumentation).

### 2.12 Detection of the level of mitochondrial apoptosis‐associated protein in tumour tissues using Western‐Blot assay

Tumour tissue was added to liquid nitrogen for grinding. After lysing 1.0 ml of RIPA lysate (Beyotime Biotechnology Co., Ltd.) on ice for 30 min, lysis was centrifuged at 10000 g for 15 min to collect the supernatant. BCA kit (Beyotime Biotechnology Co., Ltd.) was used to quantify protein concentration. After adjusting the protein concentration, Western‐Blot assay was performed according to the protocols described in the cellular experiment.

### 2.13 Enzyme‐linked immunosorbent assay for detection of inflammatory factors in peripheral blood of mice

Peripheral blood was centrifuged to collect serum centrifuged at 10,000 ***g*** for 15 min, followed by protein quantification. The assays were performed according to the manufacturer's instruction. The levels of inflammatory factors (IL‐1β, TNF‐α and IL‐6) were shown as ng/ml.

### 2.14 Statistical analysis

SPSS 17.0 software was used for statistical analysis. The experimental data were expressed as mean ± standard deviation (mean ± SD). One‐way ANOVA was used for comparison between groups, and the Bonferroni‐corrected t test was used to compare the two groups. *p* < 0.05 indicated statistical significance.

## 3 RESULTS

### 3.1 The effects of PIP on proliferation and apoptosis of HGC‐27 cells

In detecting cell proliferation rate by CCK‐8 assay, PIP was used to intervene cells at doses of 10, 20, 40 mg/L. As a result, cell proliferation rate was inhibited within 6–72 h in a dose‐dependent manner. We found that PIP IC_50_ is 25.6 mg/L through calculations. Meanwhile, the inhibition rate of cell proliferation was significantly increased along with time under the same concentration, indicating that a time‐dependent pattern of PIP on HGC‐27 proliferation inhibition. The minimum effective concentration of PIP is 10 mg/L. In terms of apoptosis, cells were treated with PIP at doses of 10, 20, 40 mg/L for 24 h, which showed significant apoptosis. In addition, the apoptotic rate increased in response to increasing dose of PIP, with significant difference among groups (*p* < 0.05). In the detection of flow cytometry, we also found that the levels of cell necrosis in the three groups were similar, but at 40mg/L, cell apoptosis was enhanced. It shows that necrosis may be dominant at low concentrations, and the level of apoptosis increases when the concentration increases.

**FIGURE 1:**
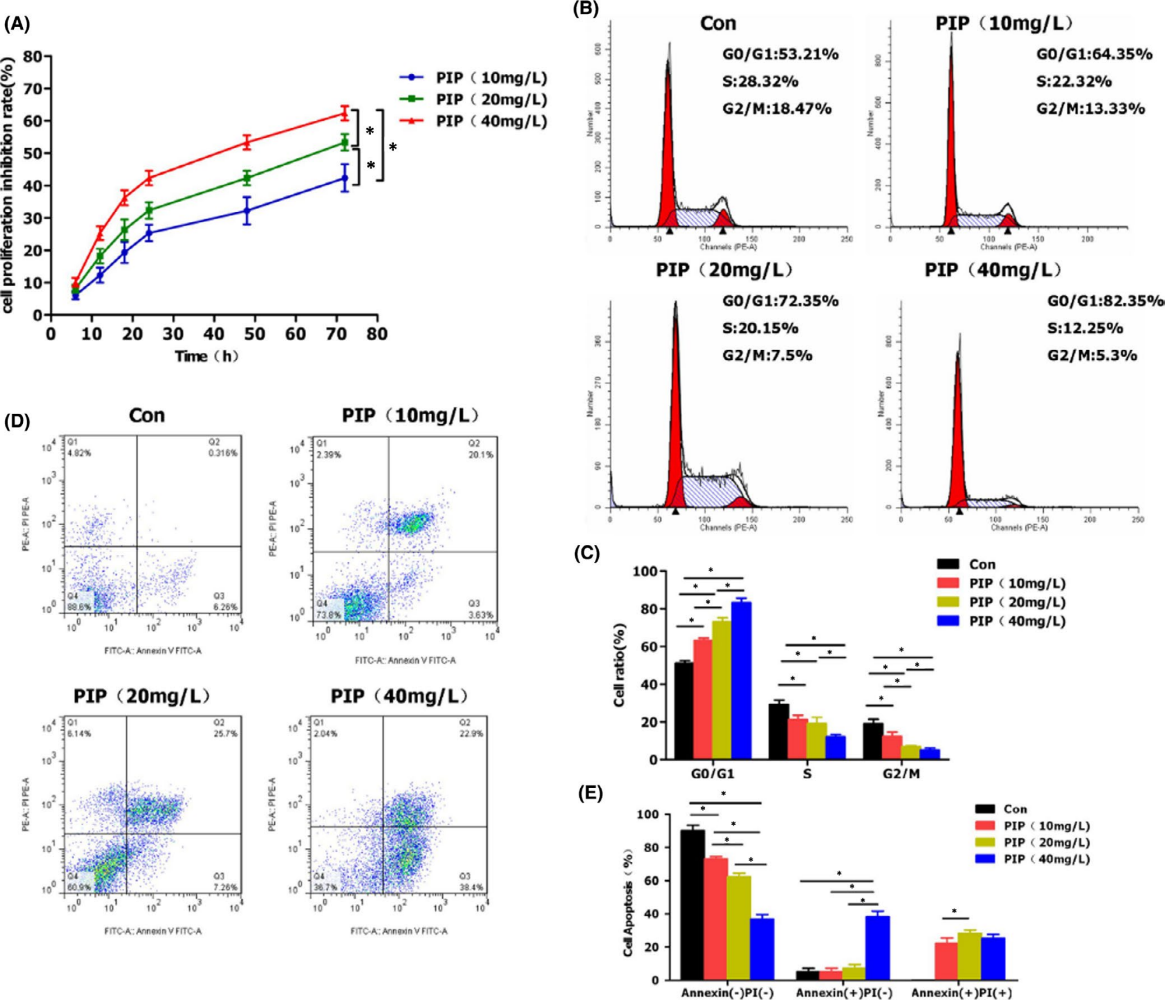
The effects of PIP on proliferation and apoptosis of HGC‐27 cells. (A) The results of inhibition rate of cell proliferation by CCK‐8 assay: After PIP treatment, the inhibition rate of cell proliferation was significantly increased along with time. And at the same time point, there was more obvious proliferation inhibition in the high‐dose group compared with the low‐dose group. Comparison between groups, *p < 0.05. (B, C) Cell cycle detection and statistical analysis: The proportion of G0/G1 phase was lower in the Con group than those in the PIP groups, while the proportion of S and G2/M phases was higher in the Con group than those in the PIP groups. In the PIP groups, the proportion of G0/G1 phase was increased, while the proportion of S and G2/M phases was decreased in response to increasing dose, showing a dose‐dependent pattern. Comparison between groups, *p < 0.05. (D, E) Apoptosis detection and statistical analysis: Annexin(‐) PI(‐) indicated normal cells, Annexin(+) PI(‐) indicated early apoptosis and Annexin(+) PI(+) suggested late apoptosis. PIP treatment could significantly increase the apoptosis rate in a dose‐dependent manner. Comparison between groups, *p < 0.05

In cell cycle analysis, the proportion of cells in the S and G2/M phases was significantly higher in the Con group compared to those in the PIP groups, while the proportion of cells in the G0/G1 phase was lower in the Con group than those in the PIP groups. Increased doses of PIP have a significant effect on cell cycle arrest, and high doses PIP can more significantly inhibit the cycle in the G0/G1 phase. The results were shown in Figure 1.

### 3.2 The effects of PIP on ROS‐mitochondria associated signals in HGC‐27 cells

**FIGURE 2:**
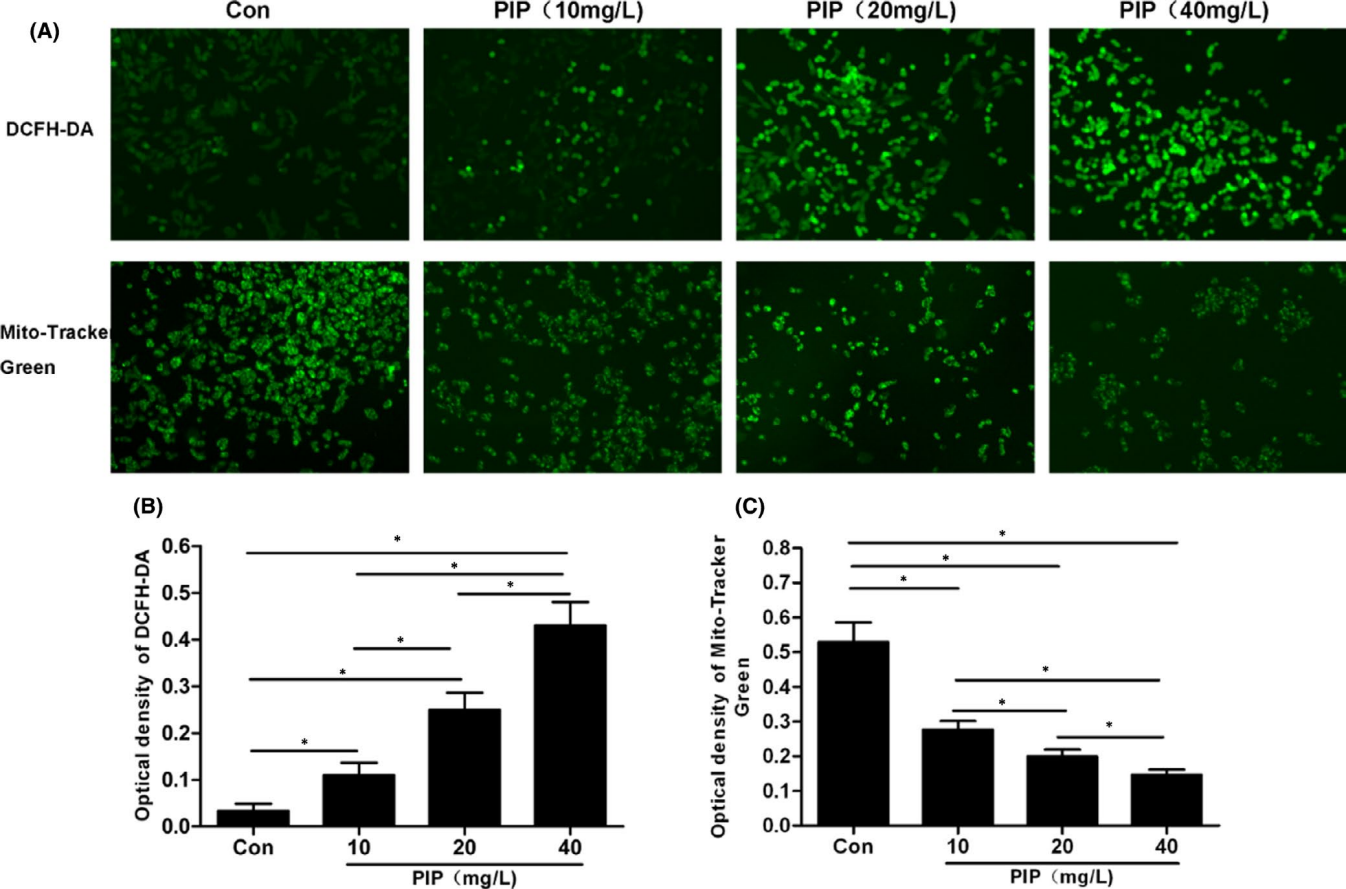
Results of the effects of PIP on ROS and viable cell mitochondria staining. (A) Fluorescence of intracellular ROS and mitochondrial staining using DCFH‐DA and Mito‐Tracker Green probes (400×). In staining using the DCFH‐DA probe, the number of positive staining cells was significantly increased after PIP treatment in a dose‐dependent manner. While using Mito‐Tracker Green probe, viable cells in the Con group were positive‐stained. PIP treatment decreased the number of positive staining cells in a dose‐dependent manner. (B) Results of fluorescence spectrophotometric detection using DCFH‐DA probe: Absorbance was relatively lower in the Con group, which was significantly increased after PIP treatment in a dose‐dependent pattern, consistent with probe detection. Comparison between groups, *p< 0.05. (C) Results of fluorescence absorbance using Mito‐Tracker Green probe: Absorbance was relatively higher in the Con group. PIP treatment caused mitochondrial damage, induced apoptosis and decreased absorbance in a dose‐dependent manner. Comparison between groups, * *p* < 0.05

**FIGURE 3:**
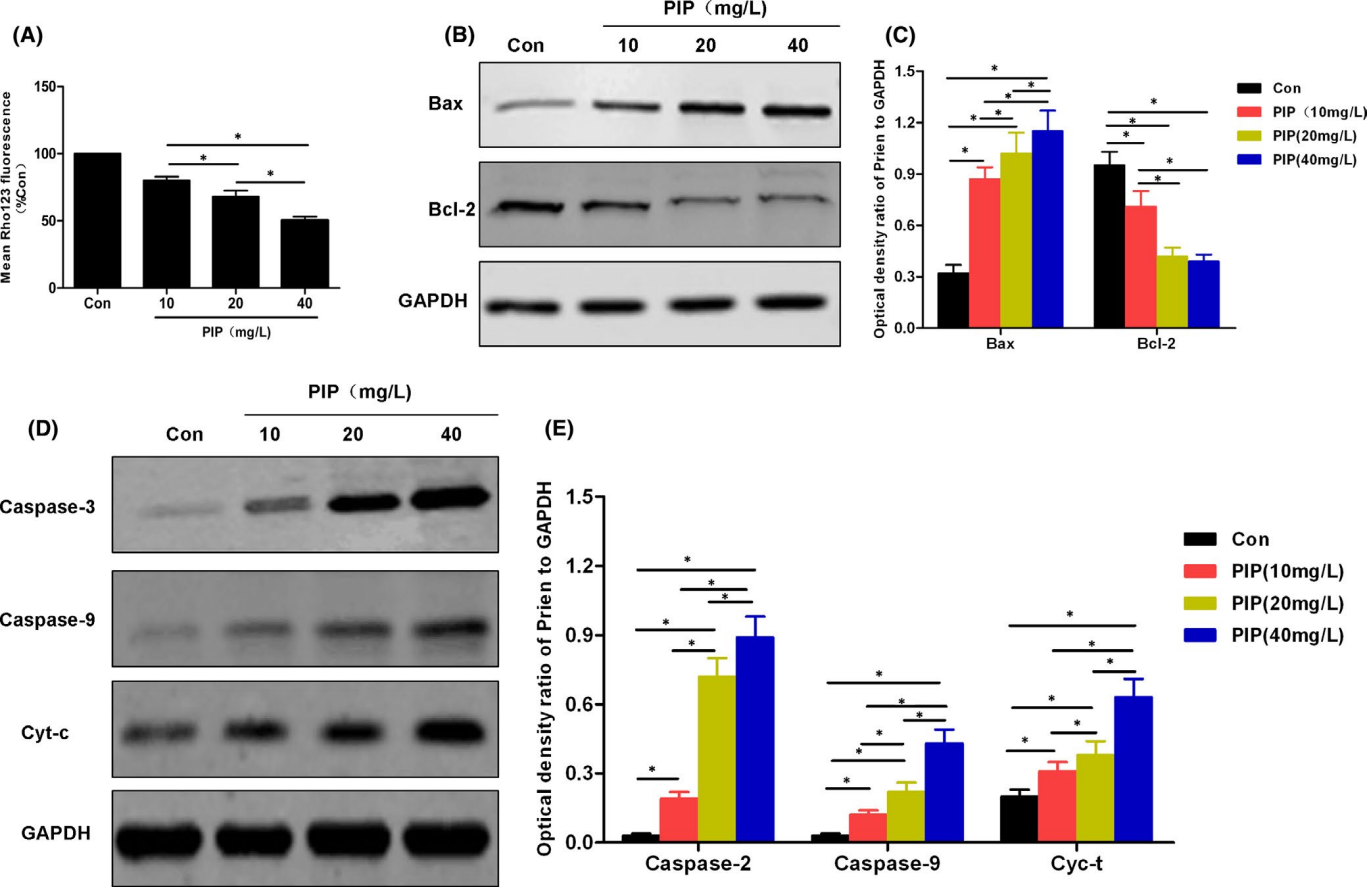
The effects of PIP on mitochondrial membrane potential and the expression of apoptosis‐associated proteins in HGC‐27. (A) Detection of mitochondrial membrane potential: Mitochondrial membrane potential was significantly downregulated after PIP treatment in a dose‐dependent manner. Comparison between groups, *p < 0.05. (B, C) Results of Bax and Bcl‐2 protein: PIP treatment significantly increased the expression of Bax and downregulated that of Bcl‐2 in a dose‐dependent manner. Comparison between groups, *p < 0.05. (D, E) Results of expression of Caspase‐3, Caspase‐9 and Cyt‐c: PIP treatment significantly downregulated the protein levels of Caspase‐3, Caspase‐9 and Cyt‐c in a dose‐dependent pattern. Comparison between groups, *p < 0.05

DCFH‐DA probe was used to detect cellular ROS generation, which showed that PIP treatment induced the generation of cellular ROS. With the increasing dose of PIP, the number of positive staining cells was significantly increased, and the value of fluorescence absorbance was enhanced, while there was no obvious ROS and low absorbance value in the Con group, with significant difference between the groups (*p* < 0.05). Mito‐Tracker Green probe was a fluorescent probe as viable intracellular mitochondrial indicator that positively stained mitochondria in viable cells, whereas in mitochondria‐injured, apoptotic cells, it was negative. PIP treatment led to decreased number of positive staining cells in cells, and reduced fluorescence absorbance in a PIP dose‐dependent pattern, with significant difference (*p* < 0.05) compared to the Con group. Rhodamine mitochondrial membrane potential assay showed that the mitochondrial membrane potential was significantly downregulated after PIP treatment in a dose‐dependent manner, which was significantly different from the Con group (*p* < 0.05). Western‐Blot assay of mitochondrial apoptosis‐associated proteins showed that PIP treatment caused downregulated expression of Bcl‐2 protein, while upregulated expression of Bax, Cyt‐c, Caspase‐3 and Caspase‐9, which was significantly different from the Con group (*p* < 0.05). The results are shown in Figures 2 and 3.

### 3.3 Intervention and mechanism of PIP on tumour‐bearing mice with gastric cancer

**FIGURE 4:**
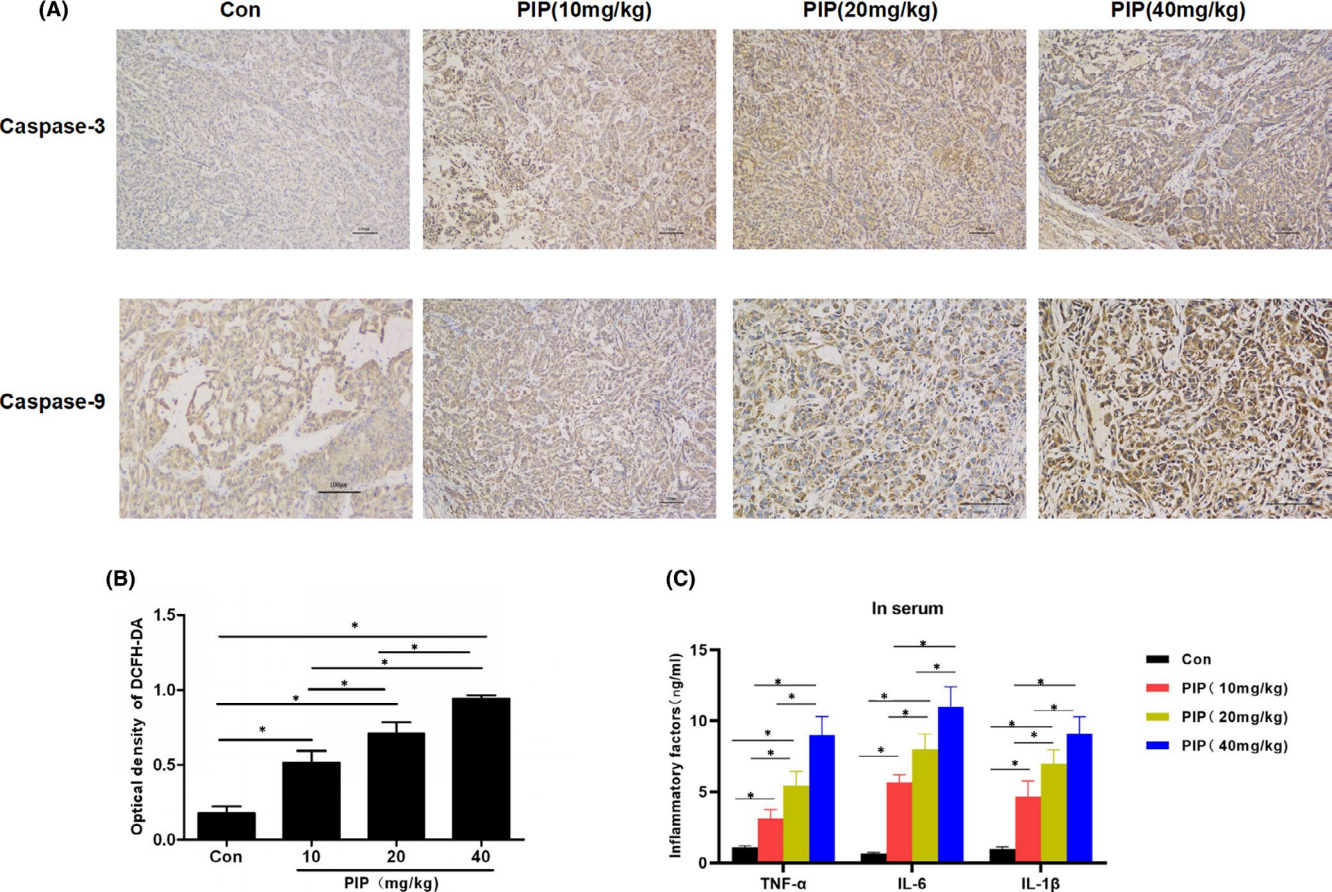
Intervention of PIP on a tumour‐bearing mouse model of gastric cancer. (A) The expression levels of Caspase‐3 and Caspase‐9 detected by IHC: Caspase‐3 and Caspase‐9 were (‐) in mice from the Con group, which were (+), (++), (+++) in mice from the 10, 20 and 40 mg/kg of PIP groups. (B) Expression of ROS in tumour tissues: Absorbance detection after ROS staining showed that absorbance was significantly increased after PIP treatment in a dose‐dependent manner, compared with the Con group. Comparison between groups was *p < 0.05. (C) The expression level of inflammatory factors: The level of inflammatory factors in the peripheral blood of the Control group mice was lower, and PIP could increase the expression of inflammatory factors in a dose‐dependent manner. Comparison between groups was *p < 0.05

**FIGURE 5:**
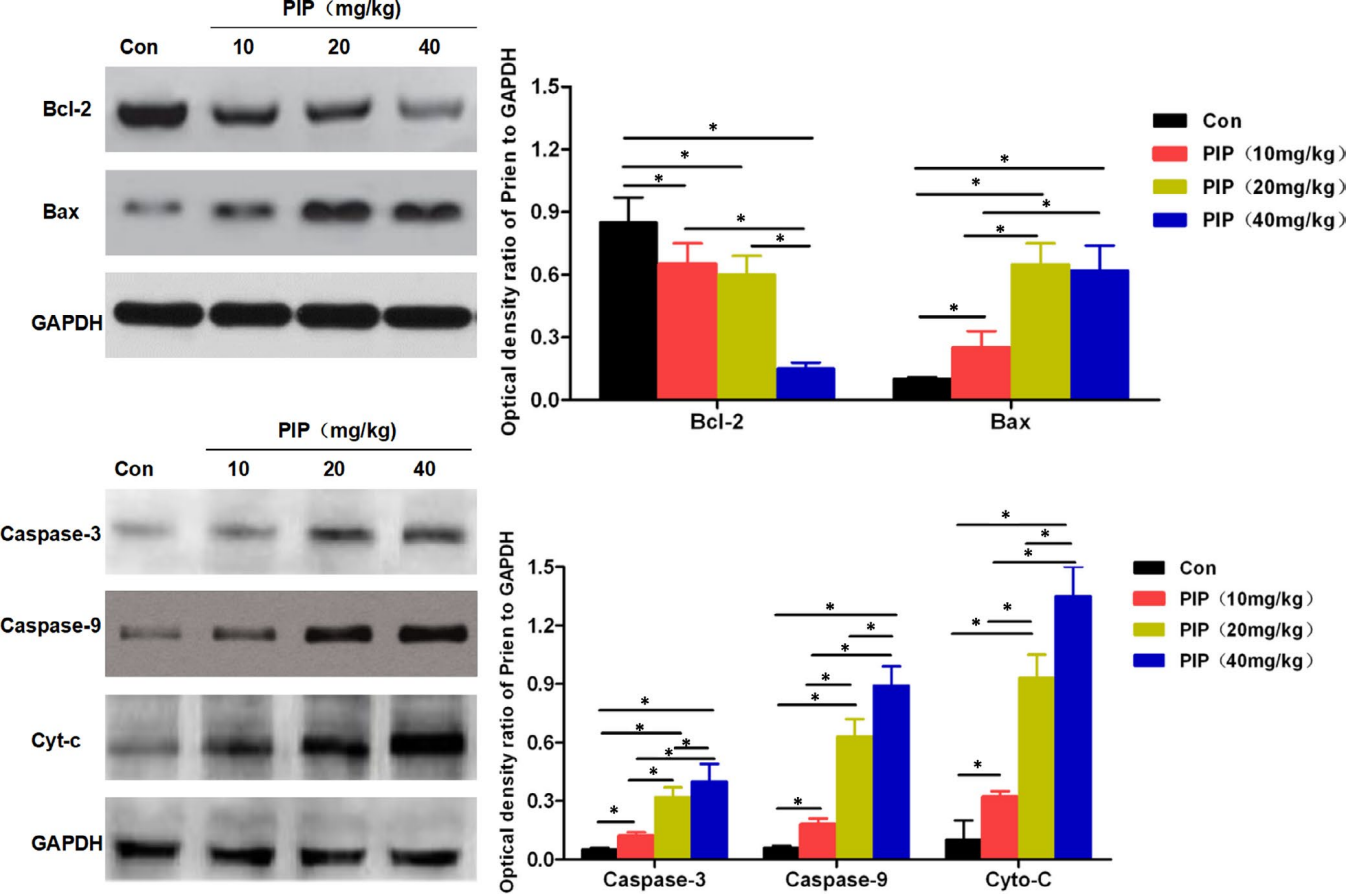
The effects of PIP on apoptotic protein in tumour‐bearing mouse model of gastric cancer. After PIP treatment, the protein level of Bcl‐2 was downregulated, while those of Bax, Cyt‐c, Caspase‐3 and Caspase‐9 were upregulated in a dose‐dependent manner. Comparison between groups, *p < 0.05

IHC staining showed that the Caspase‐3 and Caspase‐9 protein was (‐) in the Con group. After PIP treatment, Caspase‐3 and Caspase‐9 protein in tumour tissue from the three groups of mice treated with 10, 20, 40 mg/kg of PIP were (+), (++), (+++), respectively, indicating that PIP could promote apoptosis. ROS detection showed that the expression of ROS in tumour tissue was significantly increased after PIP treatment in a dose‐dependent manner, which was significantly different from the Con group (*p* < 0.05). Western‐Blot assay of mitochondrial apoptosis‐associated proteins revealed that after PIP treatment, the protein level of Bcl‐2 was downregulated, while those of Bax, Cyt‐c, Caspase‐3 and Caspase‐9 were upregulated, which was significantly different from the Con group. (*p* < 0.05). The results are shown in Figures 4 and 5.

## 4 Discussion

PIP, a cinnamic acid alkaloid, is widely distributed in nature, especially in the pepper family. Present studies have found that PIP has a wide range of pharmacological effects, including anti‐oxidation, immune regulation, anti‐tumour and promotion of drug metabolism. Of note, the effects of PIP on digestive diseases are diverse.

Apoptosis, an evolutionarily conserved mechanism, is a normal physiological process.^9^ Mitochondrial apoptosis is one of the important pathways for endogenous apoptosis. ROS, defined as a type of single‐electron reduction product,^10^, ^11^ can induce apoptosis, especially mitochondrial apoptosis.^12^, ^13^ In the study of mitochondrial apoptosis, stress damage, insectosterone compounds, inhibition of survival signals and other factors can induce apoptosis, where mitochondrial permeability transport pores (MPTP) are opened, followed by the entrance of ionic components, water, etc. into the mitochondria, causing electrochemical gradient dissipation, decreasing mitochondrial transmembrane potential, release of cytochrome C and apoptosis‐inducing factor release,^14^ further inducing cascade reaction of downstream Caspase‐3 and Caspase‐9 and, thereby inducing cell apoptosis.^15^, ^16^ ROS plays a role in all aspects, and ROS can be considered to mediate the occurrence of mitochondrial apoptosis‐associated signals. MPTP is an early event of mitochondrial apoptosis,^17^ ROS can directly interact with MPTP or induce the opening of MPTP through Bcl‐2 and Caspase family.^18^ In direct action, ROS can oxidize the sites of NADH/NAD+ and NADPH/NADP+, causing the pore structure of MPTP.^19^ Bcl‐2 is a member of apoptotic molecule family with anti‐apoptotic effect, while Bax harbours a pro‐apoptotic effect. The expression levels of Bcl‐2 and Bax affect the occurrence of apoptosis. Certain studies have found that ROS can inhibit the expression of Bcl‐2 to promote apoptosis. In downstream signals, ROS has been found to be able to activate Caspase‐9 and Caspase‐3,^20^ both of that are important proteins of the mitochondrial apoptotic pathway, playing an important role in mediating the final occurrence of apoptosis. Thus, collectively, ROS is critically involved in mediating mitochondrial apoptosis, which can induce the generation of ROS to further promote the generation of mitochondrial apoptosis. In our study, we found that PIP had a significant cytotoxic effect on gastric cancer cells. We first found that PIP‐induced necrosis of hgc‐27, which was particularly evident in low‐dose pip. With the increase of PIP concentration, the level of apoptosis was significantly upregulated. Therefore, we judge that low‐dose PIP is conducive to cell necrosis, while high‐dose pip is significant to apoptosis. Recent studies have also shown that PIP promotes the apoptosis of gastric cancer through PI3K.^21^ There are similarities with this study in the phenomenon of PIP‐induced apoptosis. Further studies have found that PIP can also inhibit cell proliferation, induce cell cycle arrest in G0/G1 phase, and significantly reduce the proportion of S and G2/M phases.

Therefore, our findings confirm that PIP inhibited proliferation by promoting apoptosis, thereby playing an anti‐cancer effect on gastric cancer. In the study mechanism, we found that PIP could promote the generation of ROS, thus, we speculated that its pro‐apoptotic effect might be related to mitochondrial apoptosis. Therefore, in‐depth studies have found that PIP treatment decreased the mitochondrial membrane potential, which was also confirmed by mitochondrial staining of viable cells. In addition, the expression of key protein of the mitochondrial apoptosis pathway, Bcl‐2, was decreased, while the expression of Bax and downstream signal, including Cyt‐c and Caspase‐3 and Caspase‐9 was increased, validating the connection of upstream and downstream cellular mitochondrial apoptotic signal after PIP treatment. In the study of gastric cancer model mice, we found that PIP can significantly activate the expression of Caspase protein, the main manifestation is that the level of Caspase‐3/9 is upregulated. Caspase‐3/9 is a key protein in the mitochondrial apoptosis pathway, which can be activated by the expression of upstream ROS.^22^ In fact, after detecting ROS in tumour tissues, we also found that PIP upregulated the level of ROS. What is more noteworthy is that PIP increases the expression of inflammatory factors. ROS is also one of the key signals of inflammatory response.^23^ ROS can activate inflammasomes such as NLRP3 to promote inflammation and necrosis of cells.^24^ We have found that ROS induced necrosis in cells, and the high expression of inflammatory factors in mice also proved the role of ROS in inducing inflammatory damage. Similarly, we also found that PIP intervention increased ROS in tumour tissue, accompanied by the decreased expression of Bcl‐2, but the increased expression of Bax, Cyt‐c, Caspase‐3 and Caspase‐9, suggesting that PIP can also activate mitochondrial apoptosis in vivo, which was consistent with cellular findings.

## 5 CONCLUSION

PIP is a chemical monomer in pepper. We found that PIP can induce apoptosis of gastric cancer cells through ROS signal. ROS has a double‐sided effect on tumour growth. Excessive ROS can induce apoptosis of tumour cells.PIP plays an anti‐tumour role by inducing ROS generation and further inducing mitochondrial apoptosis pathway, which is one of the mechanisms of anti‐tumour of PIP against gastric cancer.

## CONFLICT OF INTERESTS

No Competing interests.

## AUTHOR CONTRIBUTIONS

**Li Guo:** Conceptualization (equal); Investigation (equal). **Yi Yang:** Methodology (equal); Project administration (equal); Resources (equal). **Yongjia Sheng:** Supervision (equal); Validation (equal); Visualization (equal). **Jin Wang:** Formal analysis (equal); Investigation (equal); Methodology (equal). **Shuiliang Ruan:** Formal analysis (equal); Funding acquisition (equal); Writing‐original draft (equal); Writing‐review & editing (equal). **Chenyang Han:** Funding acquisition (equal); Writing‐original draft (equal).

## CONSENT FOR PUBLICATION

All authors’ approval are published in the article.

## Data Availability

The data and material were available.
